# Evidence-based prioritisation and enrichment of genes interacting with metformin in type 2 diabetes

**DOI:** 10.1007/s00125-017-4404-2

**Published:** 2017-08-25

**Authors:** Adem Y. Dawed, Ashfaq Ali, Kaixin Zhou, Ewan R. Pearson, Paul W. Franks

**Affiliations:** 1 Division of Molecular and Clinical Medicine, Medical Research Institute, Ninewells Hospital and Medical School, Level 5, Mailbox 12, University of Dundee, Dundee, DD1 9SY UK; 2 Department of Clinical Sciences, Genetic and Molecular Epidemiology Unit, Lund University, Skåne University Hospital Malmö, Malmö, Sweden; 3 Department of Public Health & Clinical Medicine, Umeå University, Umeå, Sweden; 4 Department of Nutrition, Harvard School of Public Health, Boston, MA USA

**Keywords:** *G6PC*, Gene-set enrichment, Metformin, *SLC2A4*, Text-mining, Type 2 diabetes

## Abstract

**Aims/hypothesis:**

There is an extensive body of literature suggesting the involvement of multiple loci in regulating the action of metformin; most findings lack replication, without which distinguishing true-positive from false-positive findings is difficult. To address this, we undertook evidence-based, multiple data integration to determine the validity of published evidence.

**Methods:**

We (1) built a database of published data on gene–metformin interactions using an automated text-mining approach (*n* = 5963 publications), (2) generated evidence scores for each reported locus, (3) from which a rank-ordered gene set was generated, and (4) determined the extent to which this gene set was enriched for glycaemic response through replication analyses in a well-powered independent genome-wide association study (GWAS) dataset from the Genetics of Diabetes and Audit Research Tayside Study (GoDARTS).

**Results:**

From the literature search, seven genes were identified that are related to the clinical outcomes of metformin. Fifteen genes were linked with either metformin pharmacokinetics or pharmacodynamics, and the expression profiles of a further 51 genes were found to be responsive to metformin. Gene-set enrichment analysis consisting of the three sets and two more composite sets derived from the above three showed no significant enrichment in four of the gene sets. However, we detected significant enrichment of genes in the least prioritised category (a gene set in which their expression is affected by metformin) with glycaemic response to metformin (*p* = 0.03). This gene set includes novel candidate genes such as *SLC2A4* (*p* = 3.24 × 10^−04^) and *G6PC* (*p* = 4.77 × 10^−04^).

**Conclusions/interpretation:**

We have described a semi-automated text-mining and evidence-scoring algorithm that facilitates the organisation and extraction of useful information about gene–drug interactions. We further validated the output of this algorithm in a drug-response GWAS dataset, providing novel candidate loci for gene–metformin interactions.

**Electronic supplementary material:**

The online version of this article (doi:10.1007/s00125-017-4404-2) contains peer-reviewed but unedited supplementary material, which is available to authorised users.

## Introduction

Metformin has been used for 60 years by more than 150 million people worldwide. It is the first-line monotherapy prescribed at diagnosis in people with type 2 diabetes [1], and also slows progression to type 2 diabetes in people with elevated but non-diabetic glucose levels who are unable or unwilling to adhere to lifestyle modification [2–4].

Despite the popularity of metformin in diabetes treatment, its mechanisms of action are poorly understood; suppression of endogenous glucose production via activation of AMP-kinase (AMPK) has been hypothesised [5]. However, a preserved glucose-lowering effect has been reported in AMPK knockout mice [6]. Alternative, non-AMPK-dependent, mechanisms include inhibition of mitochondrial glycerophosphate dehydrogenase activity [7] and adenylate cyclase-mediated inhibition of the gluconeogenic pathway in favour of glycolysis [8]. In a recent study performed on *Caenorhabditis elegans* and extended to human cell lines, Wu et al identified two new targets of metformin action: the nuclear pore complex and the gene encoding acyl-CoA dehydrogenase 10 [9].

Diabetes treatment guidelines adopt a one-size-fits-all approach, and do not take into account interindividual variation in response. Yet there is considerable between-patient variation in treatment effects, with some responding poorly or not at all and others being highly sensitive to the drug or experiencing extreme adverse drug reactions [10]. Up to 30% of individuals treated with metformin develop nausea, bloating, abdominal pain and/or diarrhoea, and 5–10% are unable to continue with metformin treatment [11]. Heritability studies indicate that genetic variation underlies around 34% of the variability in metformin response [12].

Previous candidate gene-based pharmacogenetic studies of metformin have largely focused on loci encoding transporter proteins; little emphasis has been placed on genes in the pharmacodynamics (PD) domain, and much of the published data are inconclusive and sometimes controverted [10]. Hypothesis-free genome-wide association studies (GWASs) on metformin have identified a genome-wide significant variant, rs11212617, near the *ATM* gene for metformin-induced glycaemic response [13]. Given that this SNP lies in a large block of genes that are in linkage disequilibrium, the authors performed cellular work and suggested *ATM* to be the causal gene.

AMPK, the energy sensor, is the downstream target of metformin and is believed to be involved in the PD of metformin. Selective inhibition of ataxia telangiectasia mutated (ATM) protein by KU-55933 resulted in a marked reduction in metformin-induced AMPK activation, suggesting involvement of ATM in AMPK activation. However, cellular studies showed marked inhibition of organic cation transporter (OCT)1, an important mediator of metformin uptake by the liver, by KU-55933, suggesting that the observed attenuated AMPK phosphorylation could also be due to inhibition of OCT1 [14]. A recent GWAS study from the MetGen consortium reported an association between an intronic *SLC2A2* variant, rs8192675, and the glycaemic response to metformin [15].

Owing to the vast literature on gene–metformin interactions, obtaining an unbiased overview of the evidence is extremely difficult. While meta-analysis delivers trustworthy findings if well conducted, heterogeneity in study designs, analytic strategies, population characteristics and data selection biases present challenges to such analyses [16]*.* Thus, to facilitate this process, automated approaches to integrate evidence from multiple sources, cataloguing the levels of evidence, validating in a real-world dataset, and using this to prioritise genes for follow-up are increasingly favoured [17, 18].

Here, we established a semi-automated text-mining pipeline to prioritise biological candidate genes that show evidence of interaction with metformin based on strength of evidence from published studies. We then evaluated the prioritised gene sets by examining their enrichment using a well-powered external dataset.

## Methods

### Data collection

#### Selection and download of articles

**Fig. 1:**
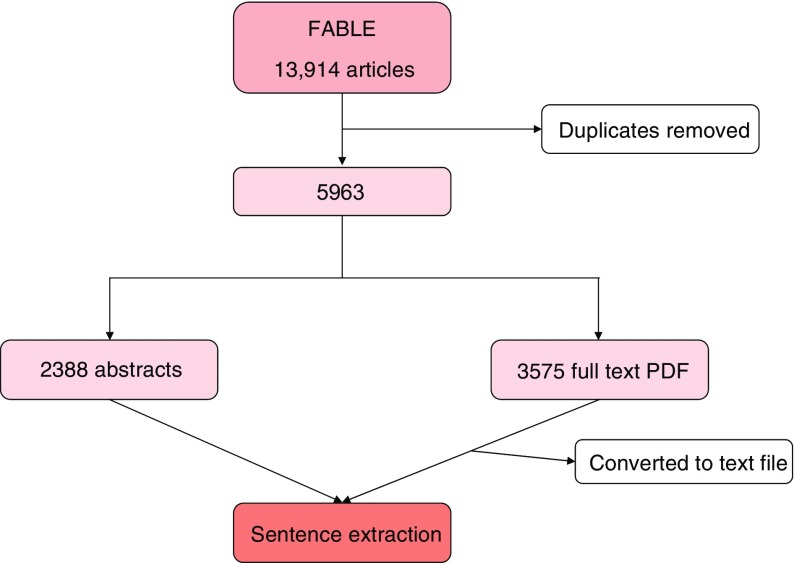
Identification, screening and selection of published articles

Articles that make reference to studies of genes and metformin in humans, identified through PubMed, were identified using the Fast Automated Biomedical Literature Extraction (FABLE) tool [19]. Accordingly, 13,914 articles were identified, of which 5963 reported independent information (Fig. 1). PubMed article identifiers (PMIDs) were collected for automated download of full text articles using Batch Entrez and EndNote. These tools permit access to articles from journals that are either open access or to which our institution (Lund University) subscribes. In most cases, PDFs are the default source of information from published articles. Thus, batch conversion of PDF to text format was done using Xpdf 3.04 (ftp.foolabs.com, accessed from 1 February to 30 June 2014).

#### Gene and drug dictionary construction

Gene and drug names are often described using more than one naming convention, abbreviation and/or synonym in the biomedical literature. Therefore, we compiled a comprehensive dictionary of gene names and abbreviations by extracting gene synonyms from NCBI Gene (www.ncbi.nlm.nih.gov/gene/), UCSC Genome Brower (www.genome.ucsc.edu/), SymAtlas (www.biogps.org/), Google (www.google.com/), GeneCards (www.genecards.org/) and iLINCS (www.ilincs.org/ilincs/), which was subsequently used to standardise data for a given gene. A drug dictionary capturing generic name, brand names, synonyms and International Union of Pure and Applied Chemistry (IUPAC) names of metformin was also developed from drug cards of the Drug Bank (www.drugbank.ca/) (see electronic supplementary material [ESM] Table 1). All these databases were accessed from 1 February to 30 June 2014.

#### Sentence extraction

Sentence extraction involves text segmentation, tokenisation and named entity recognition. Sentence segmentation and tokenisation were achieved using the Lingua::EN::Sentence module in the Perl software package, which is freely available from the Comprehensive Perl Archive Network (CPAN) (http://search.cpan.org/~shlomoy/Lingua-EN-Sentence-0.14/lib/Lingua/EN/Sentence.pm, accessed from 1 February to 30 June 2014). Gene and drug names were tagged using a Perl-based mark-up algorithm that uses a set of hashes and regular expressions. Sentences that contain a drug and a gene (i.e. a gene–drug dyad) were extracted from the corpus of each article (e.g., from titles, abstracts or main body of texts).

#### Annotation of extracted sentences

Analyses are based on the assumption that gene–drug dyads coalesce within a single sentence. Thus, each sentence was manually annotated to describe relationships between genes and metformin according to the annotation guideline given from the gene–drug interaction corpus and comparative evaluation by the Discovery through Integration and Extraction of Genomic and Clinical Knowledge (http://diego.asu.edu/, accessed 15 August 2014) [20]. ‘Interaction’ words are those that describe the presence of an interaction. For the purpose of these annotations, interactions refer to the *action*, *effect* or *influence* of the gene on a clinical outcome, pharmacokinetics (PK) or PD of the drug. Furthermore, the *action*, *effect* or *influence* of the drug on gene expression is also included as a component of *interaction*.

#### Annotation categories

Main annotations used to confirm the presence or absence of interaction between genes and metformin can be direct or indirect, and explicit or inferred. For the current analysis, three categories of data about interactions were documented: direct explicit, indirect explicit and indirect inferred. Only direct and explicit interactions were taken forward for further analysis. Different annotation subcategories were also used, along with interactions if they existed in sentences. These include ‘increased interaction’, ‘decreased interaction’ or ‘negation’. *Negation* indicates an absence of interaction and is usually represented by negative words such as ‘*not*’ or ‘*never*’ (ESM Table 2).

### Developing the evidence-scoring algorithm

An iterative ranking algorithm was developed based on the pharmacogenomic relatedness, frequency and consistency of evidence for co-occurring gene–drug pairs. Each gene was given a score based on the strength of evidence for interaction with metformin. The scoring algorithm is adapted from the Coriell Personalized Medicine Collaborative pharmacogenomics appraisal [17]. Accordingly, each gene was given a score consisting of seven categories, ranging from 1 (representing the strongest evidence; presence of consistent clinical data) to 7 (the weakest evidence; published evidence showing lack of effect of the gene on drug response). See the ESM Methods for further details.

Once all the evidence for a given gene had been gathered, a single score was assigned based on the greatest strength of evidence. For evidence scores 1–3, the drug–phenotype association should be consistent across different studies. If the data were found to be inconsistent, the clinical relevance of the gene was considered unknown and a score of 4–6, as appropriate, was returned. A score of 7 was given if the clinical relevance was clearly refutable based on the available evidence. Table 1 outlines criteria for each score with their assessment outcomes.

**Table 1:** Evidence code assignment for gene–metformin interaction

	Evidence code definition		
Evidence code	Study category	Study objective/findings	Assessment outcome
1	Clinical outcomes studies	Consistent effect of genetic variant on drug of interest^a^	Clinically relevant
2	PK or PD study	Consistent effect of genetic variant on drug of interest^a^	Clinically relevant
3	Molecular/cellular functional studies	Consistent effect of genetic variant on drug of interest^a^	Potential clinical relevance
4	Clinical outcomes studies	Inconsistent effect on drug of interest	Clinical relevance unknown
5	PK or PD study	Inconsistent effect on drug of interest	Clinical relevance unknown
6	Molecular/cellular functional studies	Inconsistent effect on drug of interest	Clinical relevance unknown
7	Clinical outcomes studies, PK or PD study	Demonstrates no effect of the genetic variant on drug response	Clinical relevance unsupported

^a^For evidence scores 1, 2 and 3, the drug–phenotype association should be consistent across different studies. If not, a score of 3–6 is assigned, as appropriate

### Genome-wide association data

#### Cohort description

The validation cohort was from the Genetics of Diabetes and Audit Research Tayside Study (GoDARTS) consisting of 2568 adults of European ancestry diagnosed with type 2 diabetes who had been on stable metformin treatment for at least 6 months with no history of insulin use before or during the study period [13]. All participants had a baseline HbA_1c_ > 7% (53 mmol/mol) and < 14% (129.5 mmol/mol).

#### Genotyping and quality control

Genotyping and quality control procedures for the GoDARTS cohort are described elsewhere [13, 14, 21]. See ESM Methods for further details.

#### Glycaemic response definition and model

A linear regression model of glycaemic response was fitted as the maximum HbA_1c_ reduction recorded within 1–18 months of the metformin index date adjusted for baseline HbA_1c_, creatinine clearance, adherence, dose, drug group and baseline gap (the number of days between baseline HbA_1c_ measure and metformin index date). The final glycaemic response model was: HbA_1c_ reduction = baseline HbA_1c_ + creatinine clearance + adherence + average daily dose + drug group + baseline gap + genotype.

Each SNP was tested for association with a continuous measure of glycaemic response (HbA_1c_ reduction) with SNPTEST v2.5 (https://mathgen.stats.ox.ac.uk/genetics_software/snptest/snptest.html) [22] using multiple linear regression assuming an additive model. Association test results were combined with fixed-effects inverse-variance-weighted meta-analysis using Genome-Wide Association Meta Analysis software v2.1.34 (www.geenivaramu.ee/en/tools/gwama) [23]. Software was accessed from 1 February to 30 June 2014.

### Gene-set enrichment analysis

We carried out enrichment analysis using Meta-Analysis Gene-set Enrichment of variaNT Associations (MAGENTA v2.4) (https://software.broadinstitute.org/mpg/magenta/, accessed from 1 February to 30 June 2014) [24] to test whether literature-identified gene sets were enriched with glycaemic response to metformin in a well-powered GWAS from the GoDARTS consisting of 2568 individuals with type 2 diabetes treated with metformin. Five sets of genes identified from the literature were used for gene-set enrichment analysis (GSEA): (1) genes directly related to clinical outcomes of metformin; (2) genes associated with either the PK or PD of metformin but not directly related to the clinical outcome; (3) genes whose expression is affected by the presence of metformin and not included in either (1) or (2) above; (4) genes related to clinical outcome, PK or PD (1 + 2); and (5) genes related to a clinical outcome and/or PK/PD/expression (1 + 2 + 3). See ESM Methods for further details.

## Results

### Data retrieval and extraction

From our screen of articles with a key word ‘metformin’ in FABLE, we identified 5963 unique articles published from 1968 to January 2014 (Fig. 1). Among these, 3575 (60%) were accessed as full text articles (ESM Fig. 1) and the remaining 2388 (40%) as abstracts (ESM Fig. 2). Although other parts may contain biologically relevant information, the best keyword per total word was obtained from abstracts [25]. ESM Fig. 3 shows the distribution of full text articles and abstracts by year of publication. A total of 2009 sentences were extracted with 3063 co-occurrences of metformin and genes. After removing non-interaction shared entities, and hypothetical, possible and indirect interactions, 1074 direct and explicit co-occurrences were annotated.

### Genes related to clinical outcomes as a consequence of metformin treatment

From the search outlined above, seven genes were identified that appear to modify the effects of metformin on diabetes-related clinical outcomes. These genes were assigned evidence code 1 and thus found to be clinically relevant (ESM Table 3). These genes included encoding proteins that affect metformin transport (*SLC22A1*, *SLC22A2*, *SLC47A1*). While *SLC22A1* and *SLC22A2* encode OCT1 and OCT2, respectively, *SLC47A1* encodes multi drug and toxin extrusion (MATE)1.

OCT1 transports metformin in the gut and facilitates its hepatic uptake [26, 27]. OCT2 and MATE1, expressed in the kidney, are widely reported to be involved in the renal excretion of metformin [28, 29]. Multiple variants in these genes are reported to affect functionality and therapeutic response to metformin [10]. *STK11*, *PRKAA2*, *ATM* and *SHBG* genes also showed consistent evidence of interactions with metformin on clinical outcomes. *ATM* encodes for serine/threonine protein kinase that belongs to the PI3/PI4-kinase family. This gene is primarily involved in DNA damage response but also involved in insulin signalling and beta cell dysfunction [30].

The liver kinase beta 1 (LKB1)–AMPK pathway controls hepatic glucose homeostasis and may play a role in the therapeutic effects of insulin-sensitising glucose-lowering agents [31]. While *STK11* encodes LKB1, *PRKAA2* encodes AMPK alpha 2 subunit. LKB1 is the upstream kinase of AMPK, an element involved in cellular metabolism and energy homeostasis [32]. Zhou et al reported that AMPK could be a key molecular effector of metformin. Activation of AMPK by metformin was shown to be associated with a subsequent reduction in the production of glucose in the liver [33]. *SHBG* encodes the sex hormone binding protein (SHBG), and variation at this locus has been related to polycystic ovary syndrome [34]. According to Ding et al, the level of circulating SHBG is inversely related to insulin resistance and may be causally related to type 2 diabetes [35].

### Genes related to PK and/or PD of metformin

Those genes that affected transport of the drug in the body or influenced metformin action but did not appear to consistently affect clinical outcomes were assigned a score of 2 (ESM Table 4). Of these, *SLC47A2*, *SLC22A3* and *SLC29A4* encode transporter proteins MATE2, OCT3 and plasma membrane monoamine transporter (PMAT), respectively. These genes were found to be predictive of the PK parameters of metformin. MATE2 is a transporter protein expressed in the brush border of the kidney [36]. Stocker et al reported an association of this protein with renal clearance and subsequent glucose-lowering effect of metformin [37]. OCT3, expressed in the brush border of the intestine and the basolateral hepatocyte membrane, could have a role in the gut absorption and hepatic intake of metformin [38, 39]. Significant interindividual variation in hepatic *OCT3* mRNA levels and association of genetic variants in *OCT3* (mRNA) with reduced *OCT3* mRNA expression in the liver has previously been reported [40]. PMAT is mainly expressed in the luminal side of the intestine and is involved in the intestinal absorption of metformin [40]. The remaining 12 genes were associated with the PD of the drug.

### Genes whose expression is influenced by metformin

Genes that encode proteins in which their cellular and molecular function is consistently affected in the presence of metformin may have potential clinical relevance. Accordingly, they were assigned a score of 3. ESM Table 5 shows a total of 51 genes that have potential relevance in predicting clinical outcome and/or PK or PD properties of the drug.

### Gene-set enrichment analysis

**Fig. 2:**
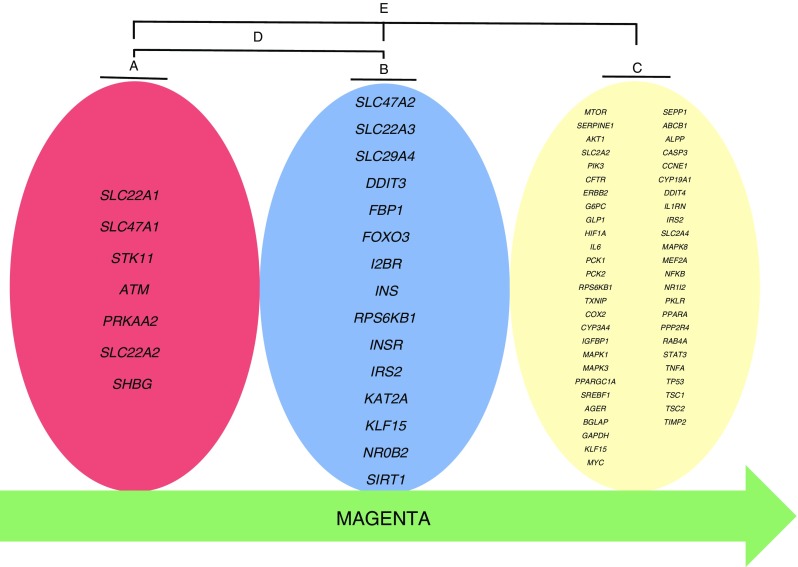
Literature-identified gene sets used for MAGENTA analysis. A, genes directly related to clinical outcomes of metformin; B, genes associated with either the PK or PD of metformin; C, genes whose expression is affected by metformin; D, genes related to the clinical outcome, PK or PD; E, genes related to clinical outcome and/or PK/PD/expression

We performed GSEA to test the enrichment of literature-identified metformin-related gene sets in a hypothesis-free GWAS from the GoDARTS. Overall, five sets of genes were constructed (Table 2 and Fig. 2) and tested for enrichment. We obtained the nominal enrichment *p* value for each gene (ESM Table 6) and then gene set after running MAGENTA (Table 3).

**Table 2:** Literature-identified gene sets used for MAGENTA analysis

Gene set	Genes
A	*SLC22A1*, *SLC47A1*, *STK11*, *ATM*, *PRKAA2*, *SLC22A2*, *SHBG*
B	*SLC47A2*, *SLC22A3*, *SLC29A4*, *DDIT3*, *FBP1*, *FOXO3*, *I2BR*, *INS*, *RPS6KB1*, *INSR*, *IRS2*, *KAT2A*, *KLF15*, *NR0B2*, *SIRT1*
C	*MTOR*, *SERPINE1*, *AKT1*, *SLC2A2*, *PIK3*, *CFTR*, *ERBB2*, *G6PC*, *GLP1*, *HIF1A*, *IL6*, *PCK1*, *PCK2*, *RPS6KB1*, *TXNIP*, *COX2*, *CYP3A4*, *IGFBP1*, *MAPK1*, *MAPK3*, *PPARGC1A*, *SREBF1*, *AGER*, *BGLAP*, *GAPDH*, *KLF15*, *MYC*, *SEPP1*, *ABCB1*, *ALPP*, *CASP3*, *CCNE1*, *CYP19A1*, *DDIT4*, *IL1RN*, *IRS2*, *SLC2A4*, *MAPK8*, *MEF2A*, *NFKB*, *NR1I2*, *PKLR*, *PPARA*, *PPP2R4*, *RAB4A*, *STAT3*, *TNFA*, *TP53*, *TSC1*, *TSC2*, *TIMP2*
D (A + B)	*SLC22A1*, *SLC47A1*, *STK11*, *ATM*, *PRKAA2*, *SLC22A2*, *SHBG*, *SLC47A2*, *SLC22A3*, *SLC29A4*, *DDIT3*, *FBP1*, *FOXO3*, *I2BR*, *INS*, *RPS6KB1*, *INSR*, *IRS2*, *KAT2A*, *KLF15*, *NR0B2*, *SIRT1*
E (A + B + C)	*SLC22A1*, *SLC47A1*, *STK11*, *ATM*, *PRKAA2*, *SLC22A2*, *SHBG*, *SLC47A2*, *SLC22A3*, *SLC29A4*, *DDIT3*, *FBP1*, *FOXO3*, *I2BR*, *INS*, *RPS6KB1*, *INSR*, *IRS2*, *KAT2A*, *KLF15*, *NR0B2*, *SIRT1*, *MTOR*, *SERPINE1*, *AKT1*, *SLC2A2*, *PIK3*, *CFTR*, *ERBB2*, *G6PC*, *GLP1*, *HIF1A*, *IL6*, *PCK1*, *PCK2*, *RPS6KB1*, *TXNIP*, *COX2*, *CYP3A4*, *IGFBP1*, *MAPK1*, *MAPK3*, *PPARGC1A*, *SREBF1*, *AGER*, *BGLAP*, *GAPDH*, *KLF15*, *MYC*, *SEPP1*, *ABCB1*, *ALPP*, *CASP3*, *CCNE1*, *CYP19A1*, *DDIT4*, *IL1RN*, *IRTK*, *SLC2A4*, *MAPK8*, *MEF2A*, *NFKB*, *NR1I2*, *PKLR*, *PPARA*, *PPP2R4*, *RAB4A*, *STAT3*, *TNFA*, *TP53*, *TSC1*, *TSC2*, *TIMP2*

A, genes directly related to clinical outcomes of metformin; B, genes associated with either the PK or PD of metformin; C, genes whose expression is affected by metformin; D, genes related to the clinical outcome, PK or PD; E, genes related to clinical outcome and/or PK/PD/expression

**Table 3:** Gene-set enrichment analysis of glycaemic response associations in literature-identified gene sets

Gene set	Nominal MAGENTA enrichment *p* value (75%)	Number of OBS genes/loci above enrichment cut-off	Number of EXP genes/loci above enrichment cut-off	Excess number of genes/loci above enrichment cut-off (OBS – EXP)	Enrichment fold (OBS/EXP)	Total number of genes
Clinically relevant genes	0.561	2	2	0	1	7
PK_PD genes	0.975	1	3			13
Gene expression	0.03	17	11	6	1.55	43
Clinically relevant + PK _PD genes	0.925	3	5			20
Clinically relevant + PK_PD + Gene expression	0.133	20	16	4	1.25	63

EXP, expected; OBS, observed

Four of the five gene sets showed no significant enrichment; the one that contained genes whose expression was affected by the presence of metformin showed significant enrichment (*p* < 0.05) (Table 3). In this gene set, six out of 17 genes above the 75th percentile enrichment cut-off (the expected number of genes above the cut-off being 11) were determined to have true associations with the glycaemic response to metformin. *SLC2A4* (*p* = 3.24 × 10^−04^), *G6PC* (*p* = 4.77 × 10^−04^) and *MAPK1* (*p* = 1.51 × 10^−03^) were among the top-ranking genes in this gene set. These genes encode GLUT4, glucose 6-phosphatase (G6PC) and mitogen-activated protein kinase 1 enzymes, respectively.

## Discussion

Patients vary greatly in their glycaemic response, optimal dosage and adverse drug reactions following metformin therapy [41]. Genetics accounts for about 34% of this variability [12]. Hence, there is a case for more personalised therapy in diseases such as type 2 diabetes. Understanding how genetic variation impacts the effects of glucose-lowering drugs or helps to refine the characterisation of type 2 diabetes might improve treatment effectiveness and decrease adverse drug reactions, morbidity, mortality and cost of treatment.

Although there are publications in relation to the PK, PD and clinical outcomes of metformin, there is no database that concisely summarises the mechanisms describing gene–drug interactions. In most cases, specific evidence of interactions is buried deep within the literature, making it extremely difficult to comprehend the overall weight of evidence for given interactions. This problem is not specific to gene–metformin interactions, but is one that is common to the gene–drug and gene–environment interaction literature per se. In this paper, however, we focus on interactions between metformin and genes that impact clinical outcomes, PK and/or PD of the drug using a comprehensive text-mining strategy.

Our analyses identified seven genes ranked as ‘top priority’ to predict metformin-related clinical outcomes. These genes constituted three solute carrier family genes (*SLC22A1*, *SLC22A2* and *SLC47A1*) that are related to the PK of metformin, and four PD-related genes (*ATM*, *STK11*, *PRKAA2* and *SHBG*). Fifteen genes were found to affect the PK/PD of metformin without being consistently related to clinical outcomes. A third gene set in which expression or activation is affected by the presence of metformin has also been identified from the text-mining. A GSEA using GWAS data from GoDARTS showed significant enrichment of the third category for glycaemic response.

Genes that showed consistent changes in cellular and molecular functions in the presence of metformin may have potential clinical relevance in the search for biomarkers that predict the therapeutic outcome of metformin. This includes *SLC2A4* and *G6PC*. *SLC2A4* encodes GLUT4, which plays a crucial role in regulating blood glucose homeostasis by facilitating insulin-stimulated glucose transport into skeletal muscles [42]. Metformin is shown to modulate translocation of GLUT4 in skeletal muscle and adipocytes [43]. *G6PC* gene encodes G6PC, a rate-limiting enzyme in hepatic glucose production. It is involved in glucose production via the gluconeogenesis and glycogenolysis pathways. Cellular studies have shown metformin to suppress glucose phosphatase enzyme expression independent of AMPK [44]. While the top genes according to the GSEA were *SLC2A4* and *G6PC*, there is a possibility that the true signal could be from neighbouring genes. ESM Fig. 4 shows regional association plots around *SLC2A4* and *G6PC*.

The stringent significance threshold for a GWAS could overlook moderate-association signals that may have detrimental collective effects in certain pathways. Therefore, integrating GSEA guided by carefully curated evidence from the literature, as we did here, is likely to identify signals that are overlooked in a GWAS. In this study, we tested enrichment of text-mining-based prioritised gene sets on a GWAS of metformin response. Gene sets that were given high priority (those related to clinical outcome, PK or PD) showed no significant enrichment for multiple modest associations. This is probably due to the fact that our scoring system is subject to the publication bias that is well known to affect candidate gene association studies. For example, there are many small studies reporting the association between variants in metformin transporter genes and glycaemic response, resulting in a higher priority of PK genes in our gene set ranking. However, a meta-analysis of most published studies, in up to 8000 metformin-treated individuals with type 2 diabetes from the MetGen consortium, showed the putative pharmacogenetics variants in five transporter genes had no significant impact on the glycaemic response to metformin [45], which is in line with our GSEA. The ranking system prioritises published evidence dominated by PK studies. Yet PK variants do not seem to alter metformin response [45]. Therefore, this is largely due to candidate gene-based approaches. The molecular and cellular-based approaches looked at mostly PD studies and hence cast the net wider, so being less focused on the transporters.

The enrichment of association signals within the low-priority gene set highlights the need to follow up association signals for loci with multiple modest effects for glycaemic response that have been previously overlooked, such as *SLC2A4* and *G6PC*, which rank high in the GWAS, with *p* values of 3.24 × 10^−04^ and 4.77 × 10^−04^, respectively.

Although the pipeline described here has generated novel evidence of gene–metformin interactions, it by no means renders a complete overview of all relevant literature. For example, only 60% of the index articles were accessible as full text, and conversion of the native PDF file into text format might have caused relevant data to be lost, as some articles used formats that are incompatible with the file templates used in our pipeline. Furthermore, data contained in tables and figures in the index papers were extracted in a semi-structured format, making data retrieved from tables and figures challenging to analyse and interpret. Thus, for papers in which important results were included in tables and figures, but not articulated in the title, abstract or main text of the article, evidence of interactions may have been overlooked.

Some genes are reported in papers in a non-standard form that may not be captured by the dictionary-based named entity recognition used in this study, and in some articles, non-standard abbreviations for metformin, instead of its original generic or brand name (e.g. the term ‘MET’), are occasionally used. Such abbreviations are not found in any standard drug abbreviation protocol. Sentences with such abbreviations are likely to be incompletely characterised using the text retrieval strategy adopted here. Other barriers to data assimilation include the extent to which anaphora (‘use of grammatical substitute [as pronoun or a pro-verb] to refer to the denotation of a preceding word or group of words’ [46]) and epiphora (‘the repetition of a word or words at the end of two or more successive verses, clauses, or sentences’ [46]) were resolved, which may also have resulted in loss of information; this might have occurred, for example, when a gene and a drug were found in separate sentences (i.e. not described in the same sentence).

Larger studies with regular iterations that use extensive literature coverage, SNP-level annotation and more intensively automated machine learning approaches are likely to extend the observations reported here. GSEA using a larger GWAS dataset for metformin response is likely to increase the power and produce convincing results. Replication using other GWAS datasets including SNP–SNP interaction analyses is also likely to extend the number of validated interactions, as some of those prioritised using our algorithm may have ethnically specific effects that are undetectable in the ethnically homogeneous GoDARTS cohort.

## Conclusions

We have developed a semi-automated text-mining and evidence-scoring algorithm that could help to organise and extract useful information from the literature on gene–drug and gene–environment interactions. According to our analysis, genes that encode transporter proteins such as OCTs and MATEs yield the strongest evidence of modifying the therapeutic outcomes of metformin. In addition, genes in the LKB1–AMPK pathway are also found to be related to the therapeutic outcomes of the glucose-lowering drug. However, we did not detect enrichment for the highly prioritised gene sets using GWAS data from the GoDARTS cohort; instead it was the gene set that were ranked with lower evidence scores that showed statistically significant enrichment in the GoDARTS.

Given that the genomic architecture of drug response is complex and the mechanism of metformin action is still not clearly known, candidate gene studies investigating drug response to metformin have had limited success. In an alternative approach, here we have identified novel genes potentially associated with metformin action. Using a text-mining approach of the published literature, we have identified a gene set derived from cellular and functional studies associated with metformin response. The association of genetic variation in these genes, including *SLC2A4* and *G6PC*, needs further replication and follow-up.

## Electronic supplementary material

ESM (PDF 552 kb)

## Abbreviations

AMPK: AMP-kinase
ATM: Ataxia telangiectasia mutated
FABLE: Fast Automated Biomedical Literature Extraction
G6PC: Glucose 6-phosphatase
GoDARTS: Genetics of Diabetes and Audit Research Tayside Study
GSEA: Gene-set enrichment analysis
GWAS: Genome-wide association study
LKB: Liver kinase beta
MAGENTA: Meta-Analysis Gene-set Enrichment of variaNT Associations
MATE: Multi drug and toxin extrusion
OCT: Organic cation transporter
PD: Pharmacodynamics
PK: Pharmacokinetics
PMAT: Plasma membrane monoamine transporter
SHBG: Sex-hormone binding protein
STK: Serine/threonine kinase
