# Supplementary material for: Evidence-based prioritisation and enrichment of genes interacting with metformin in type 2 diabetes
Source: Diabetologia. 2017 Aug 25;60(11):2231–9. doi: 10.1007/s00125-017-4404-2 (PMC6448905; doi:10.1007/s00125-017-4404-2)
Supplement: Supplementary file 1 — (PDF 552 kb) [file 125_2017_4404_MOESM1_ESM.pdf]

ESM Methods

Developing evidence scoring algorithm

Studies were categorized into three different groups:

A. *Clinical outcomes studies*. Studies within this category assess differences in clinical endpoints including but not limited to side effects, rate of cure, morbidity, and mortality. In these studies changes in the clinical outcome have been observed due to administration of metformin in the presence or absence of the mutation.

B. *PK and PD studies*. PK studies investigate the effect of a gene variant on absorption, distribution, metabolism, or excretion of the drug. Within these studies, presence or absence of the gene variant affects concentration of the drug. PD studies refer to interaction of the drug and the site of action. If a given gene variant causes differences in a biomarker in response to the drug, then it is regarded as PD type of interaction.

C. *Molecular and cellular functional studies*. Functional assays, for which there are changes in expression or activation of the gene or the respective protein or the whole cell in response to metformin, are included within this category of studies.

Genotyping and quality control

SNP genotype data on 1304 metformin treated patients were generated using the Affymetrix Genome-Wide Human SNP array 6.0 (Affymetrix, Santa Clara, CA, USA) and the remaining 1194 on Illumina HumanOminExpress (Illumina, Inc., San Diego, CA, USA). Each GWAS dataset was imputed to the 1000 Genomes CEU reference panel using the IMPUTE software [1]. Standard quality-control procedures were applied to each dataset: for example, SNPs with a minor allele frequency <5%, a call rate <98%, a Hardy-Weinburg equilibrium *p*-value $<10\times10^{-06}$, or imputation quality <40% were removed. Samples with heterozygosity >3 standard deviation from the mean or correlated with another sample (identity by descent >0.125) or markers with a high missing call rate >1% were filtered.

Gene-set enrichment analysis

GSEA was perfprmed to test whether any of the above sets of genes were represented more frequently than would be expected by chance. MAGENTA uses SNP association *p*-values and chromosome positions from the GWAS data as input. Each gene was scored based on *p*-values

among all of the SNPs located within or -110 kb upstream and +40 kb downstream from the gene. Gene scores were obtained after applying a step-wise multiple linear regression analysis corrected for gene size, number of SNPs per kb, number of independent SNPs per kb, number of recombination hotspots per kb, linkage disequilibrium units per kb and genetic distance. It tests whether the distribution of gene *p*-values in a given gene set is skewed towards low *p*-values compared with equal sized 10, 000 randomly sampled gene sets. Significant skewness below a given *p*-value cut off (enrichment cut off) suggests enrichment of the gene set. The 75th percentile *p*-value of all the genes was used as the enrichment cut-off in this analysis. The human leukocyte antigen (HLA) region was excluded from the analysis.

1. Howie BN, Donnelly P, Marchini J (2009) A flexible and accurate genotype imputation method for the next generation of genome-wide association studies. PLoS Genetics 5: e1000529.

ESM Tables and Figures

ESM Table 1. Drug dictionary with Generic names, Brand names, Chemical names and synonyms for metformin.

Chemical names	Brand names			Generic names
1,1-Dimethylbiguanide	Apo-Metformin	Glucophage XR	PMS-Metformin	Metformin hydrochloride
1,1-Dimethylbiguanide hydrochloride	Fluamine	Glumetza	Ran-Metformin	Metformin HCL
	Riomet	Glyciphage	Sandoz - metformin	
Dimethylbiguanide	Flumamine	Glycon	Ratio-Metformin	Metformin
	Fortamet	Diabefagos	Mylan-Metformin	
	Gen-Metformin	Meguan	Novo-Metformin	
	Glucophage	Diabex	Nu-Metformin	
	Diabefagos	Metiguanide	Teva-Metformin	
	Diabetosan			

ESM Table 2. Example sentences from the corpus.

PMID	Sentence	Annotation	Sub-annotation	Impact
21505202	*‘The authors concluded that ATM acts upstream of AMPK and is required for complete response to metformin.’*	DE	A	CR
21054339	*‘Metformin significantly inhibited MDR1 expression by blocking MDR1 gene transcription’*	DE	DI	GE
12130709	*‘The purpose of the present study was to investigate the role of organic cation transporter 1 (Oct1) in the disposition of metformin.’*	DE	HI	PK
8651938	*‘GLUT5 gene expression was increased by metformin treatment only in the jejunum.’*	IE	A	GE
23253948	*‘Pharmacokinetics of empagliflozin, a sodium glucose cotransporter-2 (SGLT2) inhibitor, and metformin following co-administration in healthy volunteers.’*		NISE	

DE: Direct and Explicit, IE: Indirect and Explicit, A: Affirmative effect, DI: decreased interaction, HI: Hypothetical interaction, CR: Clinical relevance, GE: Gene expression, PK: Pharmacokinetics.

ESM Table 3. Genes associated with clinical outcome of metformin.

Gene	Freq.	A	DI	N	Score
SLC22A1	26	23	1	2	1
SLC47A1	18	18	0	0	1
STK11	9	7	2	0	1
ATM	8	7	0	1	1
PRKAA2	2	2	0	0	1
SLC22A2	3	3	0	0	1
SHBG	3	0	3	0	1

A: Affirmative interaction, DI: Decreased interaction, N: No effect, Freq: Frequency of occurrence, Score: Evidence score given.

ESM Table 4. Genes associated with PK or PD of metformin.

Gene	Freq.	A	DI	N	Score
SLC47A2	17	15	0	2	2
SLC22A3	11	10	0	1	2
SLC29A4	8	8	0	0	2
DDIT3	2	2			2
FBP1	2		2		2
FOXO3	2	2			2
I2BR	2	2			2
RPS6KB1	5		4	1	3
INS	2		2		2
INSR	4	4			2
IRS2	2	2			2
KAT2A	2	2			2
KLF15	2	2			2
NR0B2	3	3			2
SIRT1	4	4			2

A: Affirmative interaction, DI: Decreased interaction, N: No effect, Freq: Frequency of occurrence, Score: Evidence score given.

ESM Table 5. Genes with potential clinical relevance.

Gene	Freq.	A	DI	N	Score
MTOR	11	1	9	1	3
SERPINE1	9		9		3
AKT1	7	2		5	3
SLC2A2	7	1	6		3
PIK3	6	1		5	3
CFTR	5		4	1	3
ERBB2	5		5		3
G6PC	5		5		3
GLP1	5	4		1	3
HIF1A	5	1	4		3
IL6	5		5		3
PCK1	5		5		3
PCK2	5		4	1	3
SLC2A4	5	5			2
TXNIP	5	1	4		3
COX2	4		3	1	3
CYP3A4	4		4		3
IGFBP1	4	3		1	3
MAPK1	4	3	1		3
MAPK3	4		3	1	3
PPARGC1A	4	4			3
SREBF1	4		3	1	3
AGER	3		3		3
BGLAP	3	3			3
GAPDH	3		3		3
KLF15	3		3		3
MYC	3	3			3
SEPP1	3		3		3

ABCB1	2		2		3
ALPP	2		2		3
CASP3	2	2			3
CCNE1	2		2		3
TIMP2	2		2		3
CYP19A1	2		2		3
DDIT4	2	2			3
IL1RN	2	2			3
IRS2	2	2			3
MAPK8	2	2			3
MEF2A	2	2			3
NFKB	2		2		3
NR1I2	2		2		3
PKLR	2	2			3
PPARA	2		2		3
PPP2R4	2	2			3
RAB4A	2	2			3
RPS6KA	2		2		3
STAT3	2		2		3
TNFA	2		2		3
TP53	2		2		3
TSC1	2	2			3
TSC2	2	2			3

A: Affirmative interaction, DI: Decreased interaction, N: No effect, Freq: Frequency of occurrence, Score: Evidence score given.

ESM Table 6. Literature identified gene *p*-values based on GoDARTS metformin related HbA1c reduction association *p*-values.

Gene symbol	MGENTA gene association *p*-value	Chromosome	Gene start Position	Gene end position	Number of SNPs per gene	Number of independent SNPs per gene	Best SNP rs number	Best SNP position	Best SNP *p*-value
ABCB1	3.84×10^{-01}	7	87133178	87342639	794	25	rs10600040	87255727	2.98×10^{-03}
AKT1	5.12×10^{-01}	14	105235686	105262080	1022	10	rs11226710	105237744	2.92×10^{-03}
ALPP	6.09×10^{-01}	2	233243347	233247599	541	13	rs112061397	233144482	8.41×10^{-03}
ATM	1.13×10^{-05}	11	108093558	108239826	506	25	rs680113	108165406	1.64×10^{-06}
BGLAP	9.45×10^{-01}	1	156211950	156213123	387	8	rs72708271	156117584	5.38×10^{-02}
CASP3	1.88×10^{-01}	4	185548849	185570629	768	14	rs78413518	185521097	1.07×10^{-03}
CCNE1	9.72×10^{-01}	19	30302900	30315219	550	18	rs147992161	30223055	6.82×10^{-02}
CFTR	6.10×10^{-01}	7	117120016	117308718	589	20	rs145128188	117141417	8.51×10^{-03}
CYP19A1	7.29×10^{-01}	15	51500253	51630795	868	39	rs7170255	51479102	8.47×10^{-03}
CYP3A4	4.13×10^{-01}	7	99354582	99381811	313	8	rs62471959	99461600	5.34×10^{-03}
DDIT3	5.02×10^{-01}	12	57910370	57914300	236	6	rs201740211	57896512	1.06×10^{-02}
DDIT4	5.74×10^{-01}	10	74033676	74035797	244	7	rs144889863	74062751	1.34×10^{-02}
ERBB2	7.47×10^{-01}	17	37844392	37884915	411	7	rs200173	37825089	2.23×10^{-02}
FBP1	4.13×10^{-01}	9	97365420	97402531	717	18	rs117307207	97430018	3.62×10^{-03}
FOXO3	7.53×10^{-01}	6	108881025	109005971	547	34	rs6913023	109037698	1.23×10^{-02}

G6PC	4.77×10^{-04}	17	41052814	41065386	147	5	rs2593595	41053567	3.61×10^{-04}
GAPDH	9.76×10^{-01}	12	6643656	6647536	510	9	rs2534718	6575191	7.62×10^{-02}
GLP1R	5.35×10^{-01}	6	39016556	39055520	664	40	rs114603402	38935443	3.84×10^{-03}
HIF1A	8.33×10^{-01}	14	62162118	62214977	573	17	rs142992098	62238358	2.22×10^{-02}
IGFBP1	4.96×10^{-01}	7	45927958	45933267	548	17	rs150899434	45942780	5.18×10^{-03}
IL1RN	6.91×10^{-01}	2	113875469	113891593	764	20	rs34645404	113781977	7.77×10^{-03}
IL6	5.73×10^{-01}	7	22766765	22771621	510	29	rs140043929	22747558	5.93×10^{-03}
INS	5.86×10^{-01}	11	2181008	2182439	664	14	rs11042982	2199963	6.85×10^{-03}
INSR	1.73×10^{-01}	19	7112265	7294011	1115	37	rs78958726	7273380	7.80×10^{-04}
IRS2	6.78×10^{-02}	13	110406183	110438914	712	26	rs9559662	110468273	2.89×10^{-04}
KLF15	4.24×10^{-01}	3	126061477	126076236	622	20	rs9814339	126026343	3.66×10^{-03}
MAPK1	1.09×10^{-01}	22	22113946	22221970	728	10	rs55741555	22076177	8.42×10^{-04}
MAPK3	9.76×10^{-01}	16	30125425	30134630	171	2	rs72791248	30195791	1.20×10^{-01}
MAPK8	2.46×10^{-01}	10	49609686	49643183	485	19	rs116987484	49672244	2.58×10^{-03}
MEF2A	1.64×10^{-01}	15	100106132	100256629	543	35	rs827796	100233972	2.36×10^{-03}
MYC	1.15×10^{-01}	8	128748314	128753680	494	22	rs117788644	128736412	7.01×10^{-04}
NFKB1	6.30×10^{-01}	4	103422485	103538459	738	28	rs72694174	103378675	8.10×10^{-03}
NFKBIA	6.30×10^{-01}	14	35870715	35873960	581	16	rs184267819	35924185	9.31×10^{-03}
NR0B2	9.51×10^{-01}	1	27237974	27240567	233	4	rs35308549	27329668	8.28×10^{-02}
NR1I2	2.10×10^{-01}	3	119499330	119537332	493	10	rs146888713	119576813:119576814	1.67×10^{-03}

PCK1	4.44×10^{-01}	20	56136136	56141513	732	47	rs79015822	56145543	2.02×10^{-03}
PCK2	5.90×10^{-01}	14	24563482	24573339	469	19	rs186277859	24605750	8.49×10^{-03}
PIK3CA	2.46×10^{-01}	3	178866310	178952497	733	17	rs193167136	178812305	1.71×10^{-03}
PKLR	3.28×10^{-01}	1	155259083	155271225	203	3	rs200273777	155245340:155245343	5.86×10^{-03}
PPARA	1.89×10^{-01}	22	46546498	46639653	1042	24	rs111300180	46605588	5.37×10^{-04}
PPARGC1A	2.45×10^{-01}	4	23793643	23891700	774	39	rs623570	23997969	1.23×10^{-03}
PPP2R4	2.65×10^{-01}	9	131873227	131911225	339	39	rs17452568	131787906	1.67×10^{-03}
PRKAA2	2.62×10^{-01}	1	57110989	57181008	886	29	rs142551250	57207265	1.43×10^{-03}
RAB4A	2.41×10^{-01}	1	229406878	229440518	546	18	rs76367805	229304470	1.68×10^{-03}
RPS6KA1	4.90×10^{-01}	1	26856248	26901520	423	12	rs114289390	26800490	6.40×10^{-03}
RPS6KB1	6.24×10^{-01}	17	57970442	58027786	413	8	rs184781576	57940064	1.19×10^{-02}
SEPP1	1.75×10^{-02}	5	42799981	42812024	545	15	rs2548366	42919122	1.42×10^{-04}
SERPINE1	6.20×10^{-01}	7	100770378	100782547	610	14	rs6961122	100663854	9.25×10^{-03}
SHBG	5.14×10^{-01}	17	7517381	7536700	589	12	rs118051719	7566428	6.63×10^{-03}
SIRT1	3.76×10^{-01}	10	69644426	69678147	537	6	rs141838640	69674786	4.09×10^{-03}
SLC22A1	1.97×10^{-03}	6	160542862	160579750	505	25	rs117143470	160575008	2.39×10^{-05}
SLC22A2	1.51×10^{-01}	6	160637793	160679963	931	29	rs477900	160622502	5.72×10^{-04}
SLC22A3	2.85×10^{-01}	6	160769424	160876014	987	31	rs117965696	160713517	1.49×10^{-03}
SLC29A4	2.26×10^{-01}	7	5322560	5343704	866	16	rs199935547	5351460	1.47×10^{-03}
SLC2A4	3.24×10^{-04}	17	7185053	7191367	318	14	rs35355120	7106038	2.46×10^{-05}

SLC47A1	9.29×10^{-01}	17	19437166	19482346	361	16	rs1808941	19451938	4.55×10^{-02}
SLC47A2	7.01×10^{-01}	17	19581627	19620043	459	18	rs140605101	19609812	1.38×10^{-02}
STAT3	8.91×10^{-01}	17	40465342	40540513	518	8	rs2354155	40546652	3.48×10^{-02}
STK11	8.61×10^{-01}	19	1205797	1228434	633	19	rs143476328	1200738	1.98×10^{-02}
TP53	5.36×10^{-01}	17	7571719	7590863	532	14	rs118051719	7566428	6.63×10^{-03}
TSC1	7.34×10^{-01}	9	135766734	135820020	701	30	rs112985725	135802090	9.90×10^{-03}
TSC2	9.75×10^{-02}	16	2097989	2138713	711	15	rs144901786	2166255	5.66×10^{-04}
TXNIP	7.52×10^{-01}	1	145438461	145442628	174	7	rs9245	145438581	2.69×10^{-02}

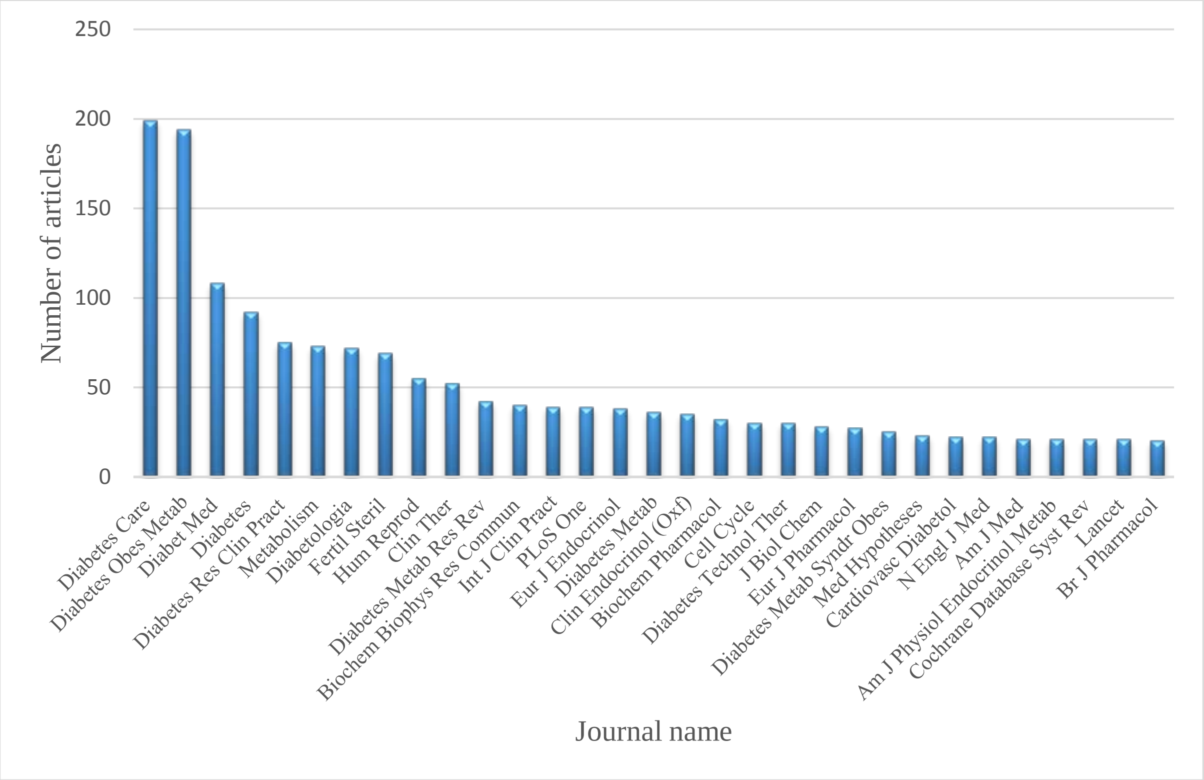

ESM Fig. 1. Frequency of full text articles downloaded by journal name.

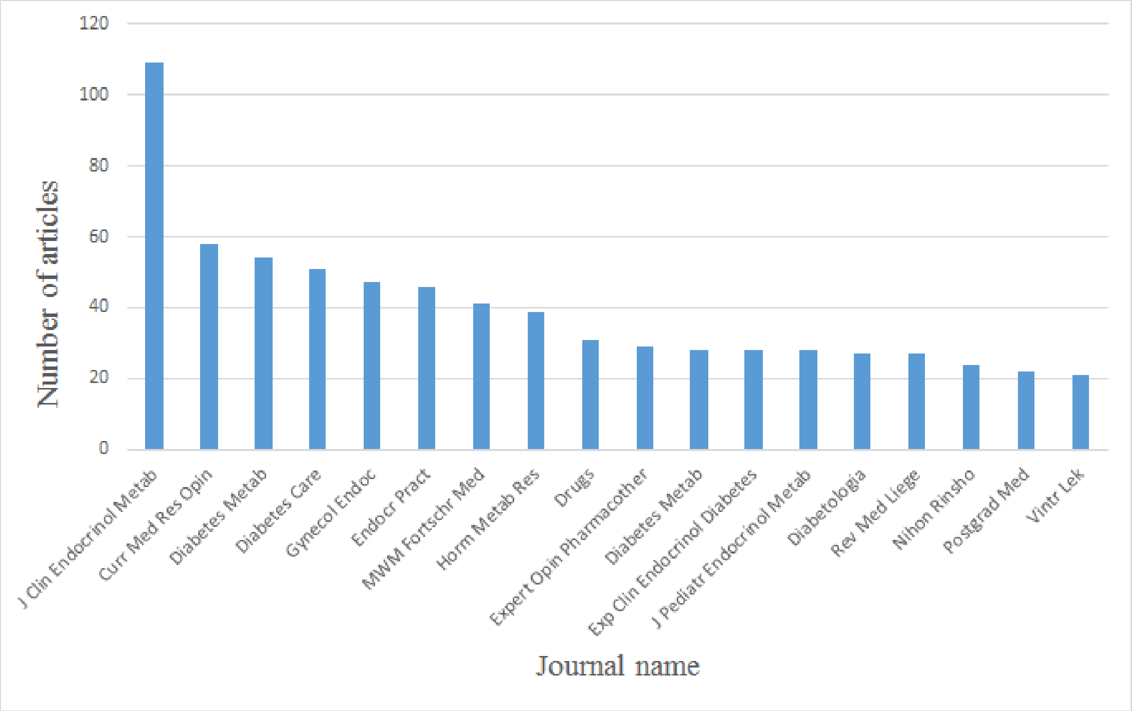

ESM Fig. 2. Frequency of abstracts downloaded by journal name.

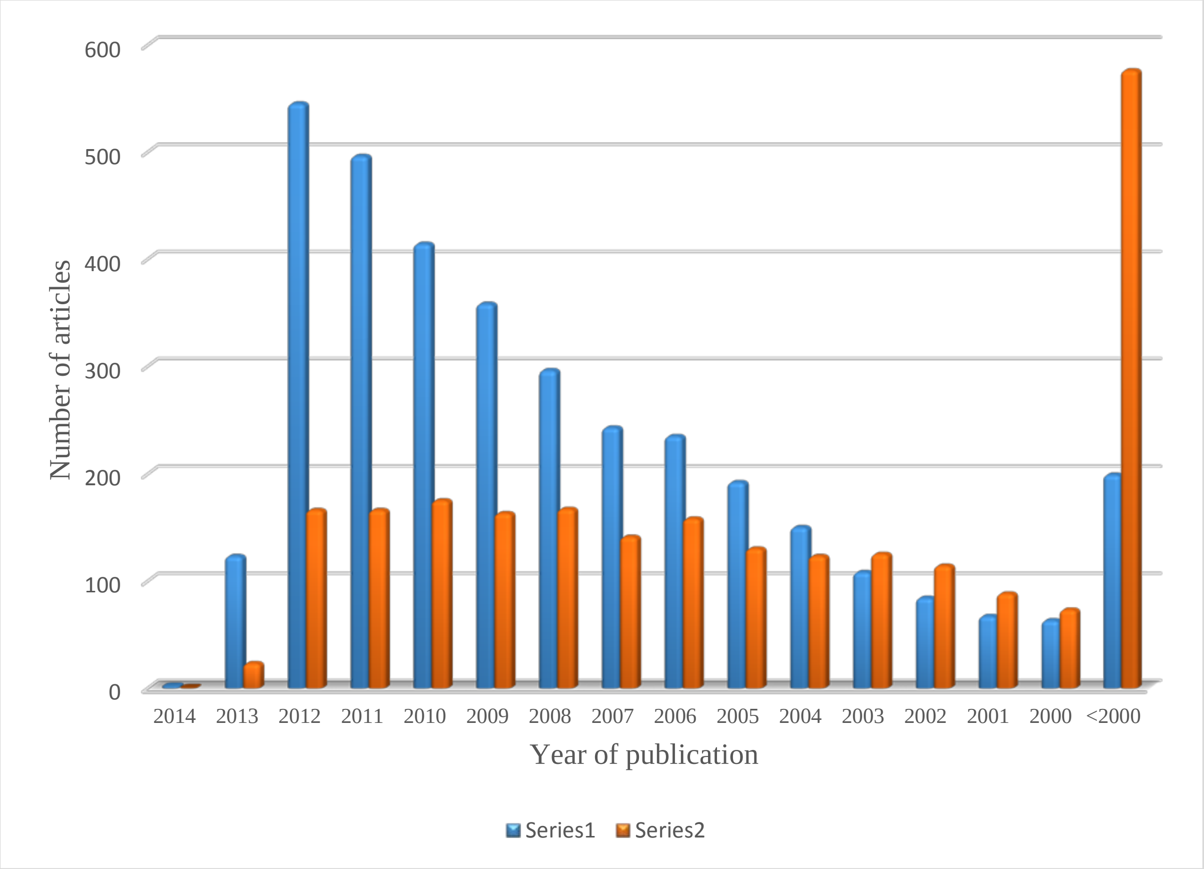

ESM Fig. 3. Article frequency by year of publication. Series 1: Full text articles, Series 2: Abstracts.

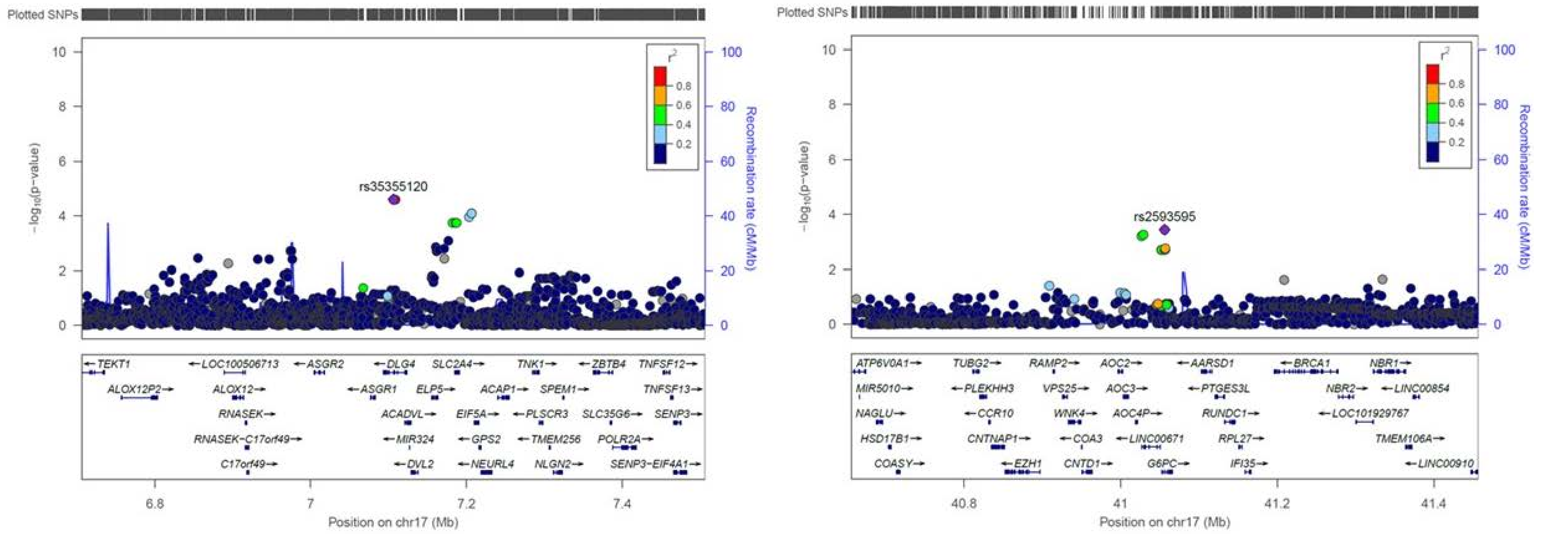

ESM Fig. 4. Regional association plots around the SLC2A4 (left) and G6PC (right) locus at chromosome 17
